# GSTT1 Copy Number Gain and ZNF Overexpression Are Predictors of Poor Response to Imatinib in Gastrointestinal Stromal Tumors

**DOI:** 10.1371/journal.pone.0077219

**Published:** 2013-10-04

**Authors:** Eui Jin Lee, Guhyun Kang, Shin Woo Kang, Kee-Taek Jang, Jeeyun Lee, Joon Oh Park, Cheol Keun Park, Tae Sung Sohn, Sung Kim, Kyoung-Mee Kim

**Affiliations:** 1 Department of Pathology, Samsung Medical Center, Sungkyunkwan University School of Medicine, Seoul, Korea; 2 Department of Internal Medicine, Samsung Medical Center, Sungkyunkwan University School of Medicine, Seoul, Korea; 3 Department of Surgery, Samsung Medical Center, Sungkyunkwan University School of Medicine, Seoul, Korea; 4 Department of Pathology, Sanggye Paik Hospital, Inje University College of Medicine, Seoul, Korea; 5 Department of Mathematics, Korea University, Seoul, Korea; University de Minho, Portugal

## Abstract

Oncogenic mutations in gastrointestinal stromal tumors (GISTs) predict prognosis and therapeutic responses to imatinib. In wild-type GISTs, the tumor-initiating events are still unknown, and wild-type GISTs are resistant to imatinib therapy. We performed an association study between copy number alterations (CNAs) identified from array CGH and gene expression analyses results for four wild-type GISTs and an imatinib-resistant PDGFRA D842V mutant GIST, and compared the results to those obtained from 27 GISTs with KIT mutations. All wild-type GISTs had multiple CNAs, and CNAs in 1p and 22q that harbor the SDHB and GSTT1 genes, respectively, correlated well with expression levels of these genes. mRNA expression levels of all SDH gene subunits were significantly lower (*P*≤0.041), whereas mRNA expression levels of VEGF (*P*=0.025), IGF1R (*P*=0.026), and ZNFs (*P*<0.05) were significantly higher in GISTs with wild-type/PDGFRA D842V mutations than GISTs with KIT mutations. qRT-PCR validation of the GSTT1 results in this cohort and 11 additional malignant GISTs showed a significant increase in the frequency of GSTT1 CN gain and increased mRNA expression of GSTT1 in wild-type/PDGFRA D842V GISTs than KIT-mutant GISTs (*P*=0.033). Surprisingly, all four malignant GISTs with KIT exon 11 deletion mutations with primary resistance to imatinib had an increased GSTT1 CN and mRNA expression level of GSTT1. Increased mRNA expression of GSTT1 and ZNF could be predictors of a poor response to imatinib. Our integrative approach reveals that for patients with wild-type (or imatinib-resistant) GISTs, attempts to target VEGFRs and IGF1R may be reasonable options.

## Introduction

Gastrointestinal stromal tumors (GISTs) are the most common mesenchymal tumor of the gastrointestinal tract with an annual incidence ranging from 11 to 19.6 per million population, which corresponds to between 3,300 and 6,000 new cases per year in the United States [1]. The gold standard for treating a localized primary GIST is surgical resection [2]. However, tumor recurrence is common and usually occurs in the liver and/or the peritoneum [3]. GISTs have received considerable attention due to their sensitivity to tyrosine kinase inhibitors.

Oncogenic KIT and PDGFRA mutations in GISTs correlate with tumor phenotype, prognosis, and therapeutic responses to tyrosine kinase inhibitors [4,5]. However, kinase mutation status does not fully explain the complex biology of GISTs. Moreover, approximately 85% of pediatric GISTs and 10-15% of adult GISTs do not harbor mutations of KIT or PDGFRA genes (so called ‘wild-type’ GISTs) [6–8]. Although mutations in BRAF, RAS, and the succinate dehydrogenase (SDH) subunits have recently been identified in a subset of these tumors, the tumor-initiating events in wild-type GISTs are still not fully understood [1]. Moreover, wild-type GISTs are less sensitive to imatinib than GISTs harboring mutations in exon 11 of KIT gene. This may in part be due to differences in the ability of imatinib to inhibit wild-type versus mutant forms of KIT, but there may be other underlying mechanisms that could be uncovered by high-throughput approaches [9–11]. In the future, patients with progressive disease may be preselected for treatment with imatinib or alternative and/or additional therapies based on their KIT/PDGFRA mutational status and predictive gene signatures of drug response [12].

Previous comparative genomic hybridization (CGH) studies have shown frequent loss of 14q, 22q, 1p, and 9p (including the genes PARP2, APEX1, NDRG2, SIVA, NF2, ENO1 and CDKN2A/2B), and gain of 8q (including MYC) [13]. Array-based studies have also demonstrated site-dependent chromosomal imbalances in GISTs, indicating that frequent losses at 14q are associated with gastric GISTs and losses of 1p are related to intestinal GISTs and an aggressive clinical course [14–16]. However, all previous studies focused on KIT-mutant GISTs, and no studies on wild-type GISTs have been reported. To explore potential target genes or mechanisms underlying imatinib resistance in wild-type GISTs, we integrated CGH and expression profiling in 32 gastric GISTs, including four wild-type GISTs and one imatinib-resistant PDGFRA D842V mutant GIST.

## Materials and Methods

### Case selection

Written informed consent was obtained from all patients, and the present study was conducted after the approval from the Institutional Review Board of Samsung Medical Center. Thirty-two cases of primary gastric GIST were selected based on the availability of fresh-frozen tissue among patients who had undergone complete surgical resection (R0) at the institution between 2006 and 2010. Eighteen patients were male and 14 were female with ages ranging from 34 to 81 years (mean, 62 years). Four risk groups stratified by tumor size and mitotic counts comprised eight cases each.

The presence of mutations in exons 9, 11, 13, and 17 of KIT and exons 12, 14, and 18 of PDGRFA were investigated by sequencing these exons using an ABI 3700 automated sequencer (Applied Biosystems, Foster City, CA, USA) as described previously [17]. KIT exon 11 mutations were observed in 26 cases (81%), and consisted of 10 deletion, 5 duplication/insertion, and 11 missense mutations. One case each harbored a missense mutation in KIT exon 17 (N822K) and PDGFRA exon 18 (D842V). The remaining four (13%) GISTs were wild-type for both KIT and PDGFRA genes.

### Array comparative genomic hybridization

Array comparative genomic hybridization (aCGH) was performed on the 32 cases using the Agilent Human Genome CGH Microarray Kit 244K (Agilent Technologies, Santa Clara, CA, USA). We followed the procedures for DNA digestion, labeling, and hybridization described in Agilent’s protocol version 4.0. A pool of normal genomic DNA (Promega, Madison, WI, USA) was used as a reference according to the patient’s gender. Data were obtained using Agilent feature extraction software and analyzed with Agilent Genomic Workbench version 6.0 software using the ADM-2 algorithm with a sensitivity threshold of 6.0 and a moving average window of 2 Mb or 20 Kb. Minimal overlapping regions of gain and loss were determined by assessing the smallest alteration regions identified in three or more of the samples [18]. The copy number (CN) data are available in Gene Expression Omnibus (GEO) with the accession number GSE47912.

### Gene expression profiling

Gene expression analysis was carried out using the Agilent 44K Human Gene Expression Array that contains over 41,000 human genes and transcripts. Total mRNAs were extracted from 15 fresh tissues of three wild-type, one PDGFRA-mutant, and 11 KIT-mutant GISTs using the RNeasy Mini Kit (Qiagen, Valencia, CA, USA). RNAs (~200 ng) were reverse-transcribed into cDNAs and quantiﬁed using a NanoDrop ND-1000 (Thermo, Fisher Scientiﬁc, Waltham, MA, USA). A reference pool was made by combining equal amounts of RNAs from three schwannomas and four leiomyomas from the stomach. The microarray was hybridized using an Agilent SureHyb chamber and incubated in a Rotisserie hybridization oven. Slides were washed in Gene Expression Wash Buffers and then scanned on an Agilent microarray scanner. Data were extracted with Agilent feature extraction software and normalized by quantile and VSN (variance stabilizing normalization) method using Agilent Genespring GX software 11.5 and Bioconductor packages, respectively. Differences in expression level between wild-type/PDGFRA-mutant and KIT-mutant GISTs were calculated using the Mann-Whitney U test. The *P* values were adjusted using the Benjamini and Hochberg false discovery rate method. Differentially expressed genes were selected based on ≥10 fold change with *P* value <0.001 and ≥2 fold change with *P* value <0.05 for quantile and VSN normalized data, respectively. The expression data are also available in GEO under the accession number GSE47911.

### Quantitative real-time PCR

Genomic DNA and total mRNA were extracted from 43 formalin-fixed, paraffin-embedded GISTs using the QIAamp DNA Mini Kit and RNeasy FFPE Kit (Qiagen). The CN of GSTT1 (Assay ID Hs00659429_cn) was determined using the Taqman Copy Number Assay (Applied Biosystems). Total RNA was reverse transcribed using the High Capacity cDNA Reverse Transcription Kit (Applied Biosystems) and then mRNA expression of GSTT1 (Assay ID Hs01091675_g1) was quantified using the Taqman Gene Expression Assay (Applied Biosystems). RNase P was used as an endogenous reference control for gene CN and the ACTB gene was used as an endogenous reference control for mRNA expression analyses. Reactions were performed using the 7900HT Fast Real-Time PCR System (Applied Biosystems) in quadruplicate, and relative quantity was calculated by the 2^-∆∆Ct^ method.

### Loss of heterozygosity (LOH) analysis

Multiplex PCR-amplification of microsatellite sequences was performed to determine LOH in the SDHB gene. Forward primers were end-labeled with florescent dyes (6-FAM, VIC and PET; Applied Biosystems). Each PCR reaction contained 100 ng of DNA, 10-50 pmol of fluorescent-labeled forward primer and unlabeled reverse primer, and 17 µl of PCR premix (iNtRON Biotechnology, Korea). PCR products were diluted with 20 µl of H_2_O, and 2 µl of the dilution was combined with 10 µl of Hi-Di formamide and 1 µl of Genescan 500 LIZ size standard (Applied Biosystems). Samples were capillary electrophoresed on an ABI 3130xl and analyzed using Genescan analysis software version 4.0 (Applied Biosystems). LOH was defined as a reduction of at least 50% in the allelic ratio between the tumor and normal DNA from the same patient, while homozygosity was classified as noninformative.

## Results

### Gene copy number alterations

**Figure 1:**
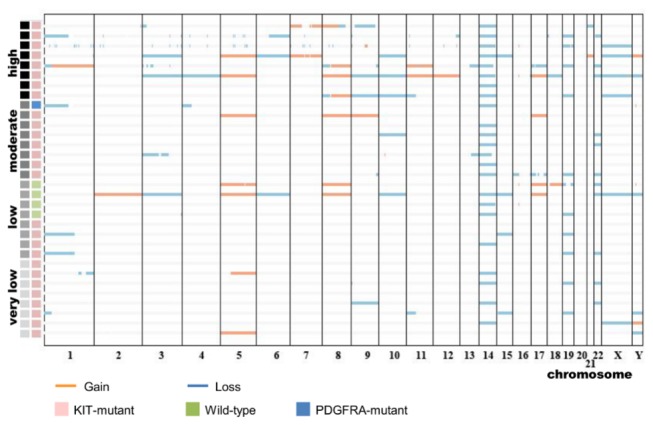
Distribution of copy number alterations in 32 gastrointestinal stromal tumors.

In total, 1138 copy number alterations (CNAs) were detected in the 32 GIST samples and the mean number of CNAs per patient was 35.6 (range, 7-129). There was a mean of 51.7 aberrations per chromosome (range, 14-115), and deletions outnumbered amplifications by over two-fold. Of the CNAs, frequently lost regions were on chromosomes 1q, 16q, 14q, 3q, 17q, 4q, 6p, and 22q, whereas regions commonly gained were on chromosomes 8p, 1q, 7q, 11q, 15q, 16q, 5p, and 1p. There were no significant differences in the number of CNAs between mutation types (wild-type vs. KIT/PDGFRA mutations) or among prognostic risk subgroups. The clinicopathologic data of these 32 gastric GISTs and the CNAs detected by aCGH are shown in Figure 1 and Table S1.

### Identification of differentially expressed genes

**Figure 2:**
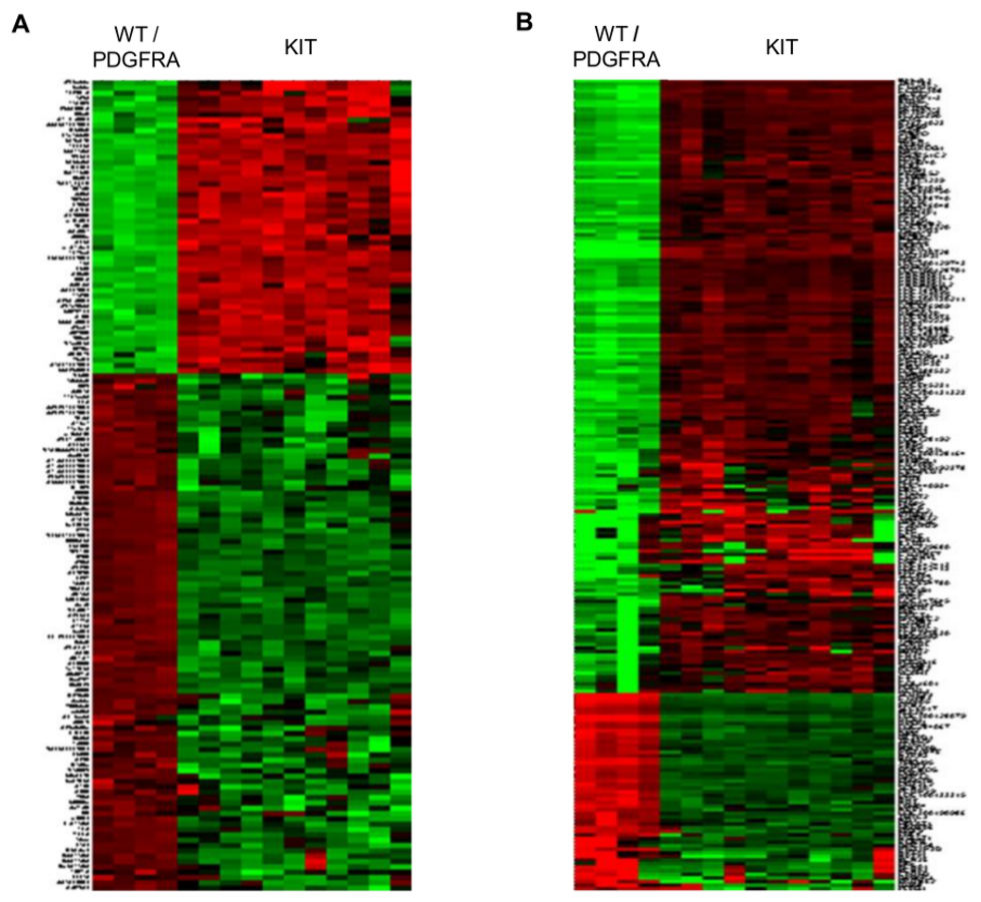
Heat map of differentially expressed genes after quantile (A) and VSN (B) normalization.

The relation between tumor genotype and gene expression profile was analyzed for 15 GISTs; the unsupervised hierarchical clustering results are shown in Figure 2. Three wild-type GISTs and a PDGFRA D842V GIST formed a tight cluster on two distinct dendrogram branches. To identify genes differentially expressed between wild-type/PDGFRA-mutant and KIT-mutant GISTs, we applied two different normalization methods, and 60 commonly shared genes were identified in both analyses (34 underexpressed and 26 overexpressed genes). Functional annotation analysis was performed using DAVID bioinformatics resources, and Table 1 shows a list of the top-ranked categories based on gene ontology (GO).

**Table 1:** Top functional annotation terms of the 60 differentially expressed genes.

	Terms	Ratio (%)	*P* value	Benjamini	Genes
Overexpressed (n=26)	GO:0031224 ~ intrinsic to membrane	50.0	0.010	0.363	*TMEM95, **NRP1**, SCN3A, PCDH19, FAM57B, NTRK3, **SSTR3**, UCP3, GIPR, DPEP3, TMTC3, IGSF9, GPR12*
	GO:0030516 ~ regulation of axon extension	7.7	0.023	0.995	*NTRK3, **NRP1***
	GO:0016021 ~ integral to membrane	46.2	0.025	0.454	*NTRK3, **NRP1**, TMEM95, **SSTR3**, UCP3, SCN3A, GIPR, TMTC3, IGSF9, PCDH19, GPR12, FAM57B*
	GO:0019932 ~ second-messenger-mediated signaling	11.5	0.031	0.971	*SSTR3, GIPR, HIST1H4E*
	GO:0016358 ~ dendrite development	7.7	0.041	0.957	***NRP1**, IGSF9*
Underexpressed (n=34)	GO:0034728 ~ nucleosome organization	8.8	0.006	0.371	***SET**, NAP1L1, HIST1H3I*
	GO:0006325 ~ chromatin organization	11.8	0.011	0.388	***SET**, MORF4, NAP1L1, HIST1H3I*
	GO:0006350 ~ transcription	20.6	0.037	0.729	*SCRT2, NACC1, PTGER3, MORF4, FOXC2, NFE2L1, SOX8*
	GO:0065003 ~ macromolecular complex assembly	11.8	0.048	0.784	*NACC1, **SET**, NAP1L1, HIST1H3I*
	GO:0008009 ~ chemokine activity	5.9	0.055	0.935	*CXCL5, CKLF*

Bold font indicates cancer-related genes according to the Ingenuity knowledge database.

**Figure 3:**
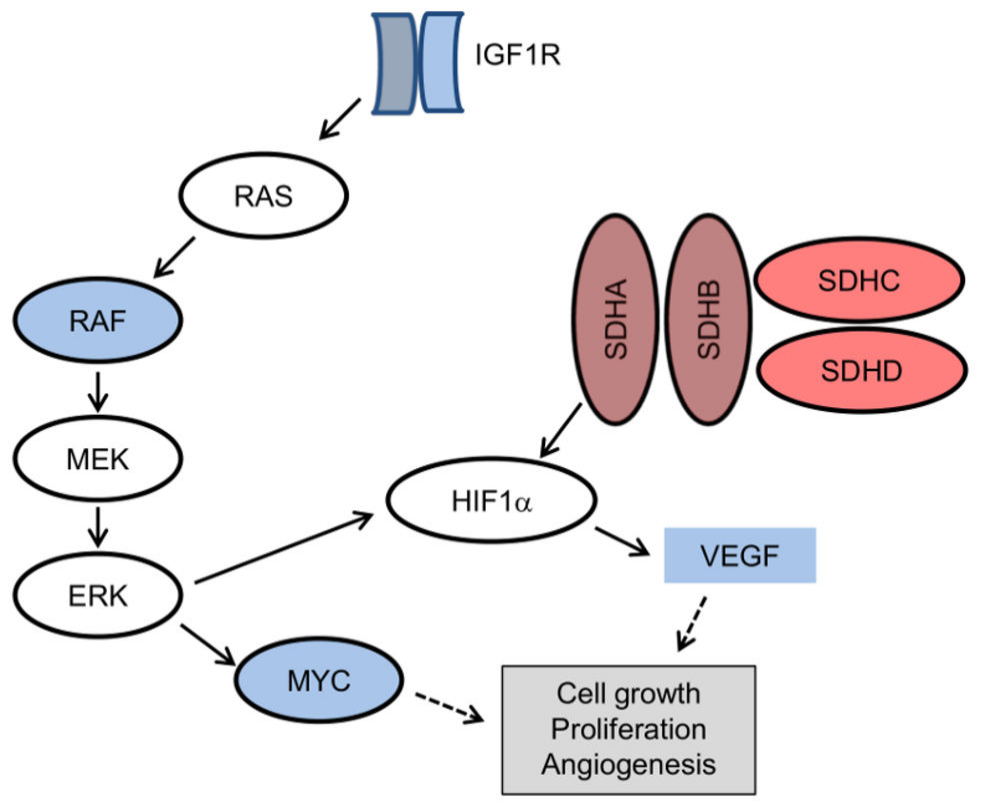
Altered mRNA levels in wild-type/PDGFRA-mutant gastrointestinal stromal tumors (red, decreased; blue, increased).

In addition, several genes known to be involved in important oncogenic signaling pathways or those associated with malignancy in GISTs (reviewed in Ref. 1) were differentially expressed between the two groups. The quantile-normalized fold change values are listed in Table S2. Among them, the mRNA levels of all SDH subunits (A, B, C, and D) were significantly lower (0.85 to 3.23-fold decrease, *P*≤0.041), whereas the expression of VEGF (2.31-fold increase, *P*=0.025) and IGF1R (2.76-fold increase, *P*=0.026) was higher in wild-type/PDGFRA D842V GISTs than in KIT-mutant tumors. Among constituents of the MAPK cascade, mRNA levels of BRAF (0.50-fold increase, *P*=0.001) and its downstream effector, MYC (2.21-fold increase, *P*=0.017), were also increased in wild-type/PDGFRA D842V GISTs than in KIT-mutant GISTs. These gene expression results are also summarized in a schematic diagram (Figure 3). Additionally, of 39 zinc finger (ZNF) genes mapped to 19p12-13.1, 32 (82.1%) were more highly expressed in wild-type/PDGFRA-mutant GISTs than in KIT-mutant GISTs (0.49 to 4.08-fold increase, *P*<0.05).

### Integration of genomic copy number results with expression profiles

**Figure 4:**
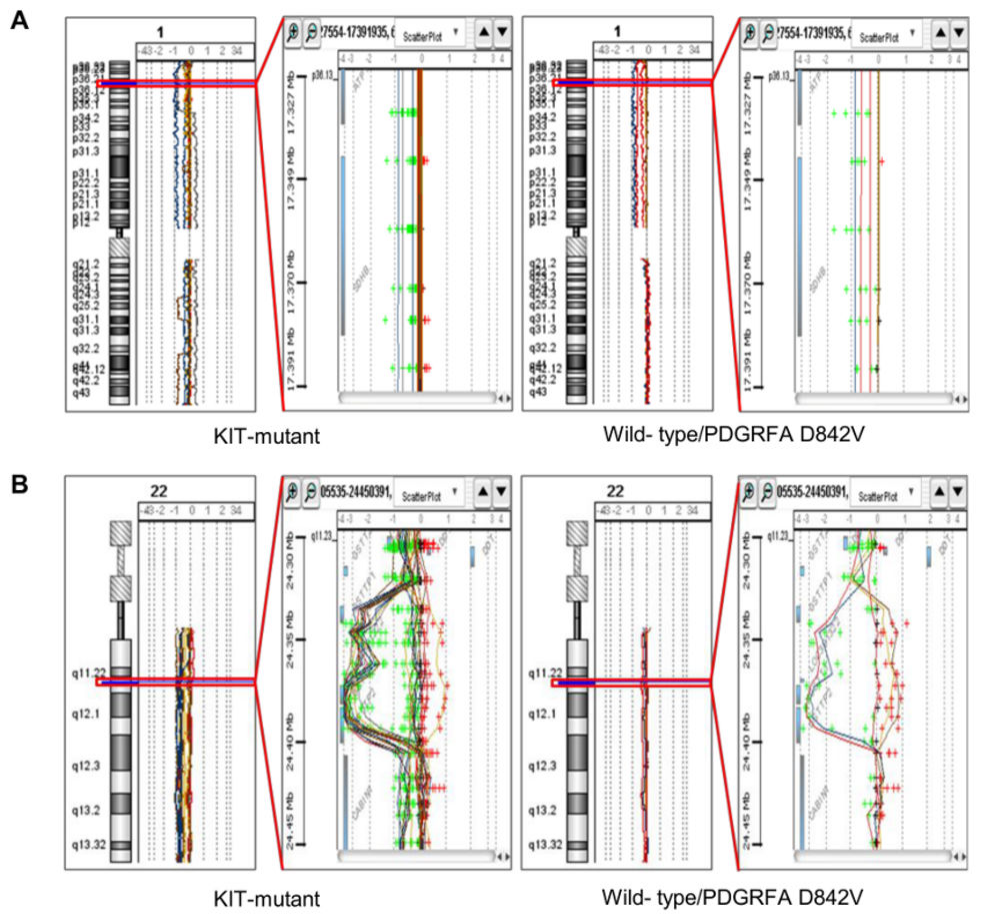
Representative photographs of high-resolution comparative genomic hybridization (A, SDHB locus; B, GSTT1 locus).

To integrate genomic CN data with expression profiles, we first identified 20 minimal overlapping regions that were gained or lost in three or more (60%) of five patients with wild-type/PDGFRA-mutant GISTs based on aCGH data (Table 2). Recurrent losses and gains were observed in 15 and five regions, respectively. When all genes on the arrays were grouped based on their chromosomal locations, the 20 loci included more than 2,800 genes represented by 41,091 probe sets in the Agilent gene expression array. These regions also contained some of the genes implicated in the development or progression of GISTs, such as mTOR, JUN, NRAS, SDHB, SHC, SMARCA3, HSP90, SIVA, STAT3, TOP2A, and GRB2 (reviewed in Ref. 1). Two representative chromosomal loci found by aCGH at high resolution are shown in Figure 4.

**Table 2:** Minimal overlapping regions in wild-type/PDGFRA-mutant gastrointestinal stromal tumors and the corresponding genes.

				Differentially expressed genes (n=37)		
Loss (n=15)		Previously identified genes		>2.5 fold decrease		*P*<0.0001
14q13.1 - q13.2		·		·		·
14q32.33		*HSP90, SIVA*		·		·
16p13.11		·		·		·
16q22.1		·		·		*THAP11, NUTF2, COG8*
17q21.31		*STAT3, TOP2A*		*KRTAP1-3, KRTAP4-1*		*CASC3*
17q25.3		*GRB2*		·		*GRB2, SLC26A11*
1p36.33 - p11.2		*mTOR, JUN, NRAS, SDHB*		*CYP2J2, CRYZ, ECHDC2, HOOK1, PTGER3, DFFB, CDC14A, **VCAM1**, ICMT, HNRNPCL1*		*DVL1, **TARDBP**, ICMT, NADK, CLIC4, ARTN, GPSM2*
1q21.3		*SHC1*		*LCE2D*		*ZNF687, LCE2D, SPRR3*
1q42.13		·		*DUSP5P*		*ARF1, TRIM11*
20p13		·		*SCRT2*		·
19q13.31		·		·		*TEAD2*
3q26.1		*SMARCA3*		*SLITRK3, BCHE*		·
4q13.2		·		·		·
6p21.32		·		·		·
9q34.3		·		·		*ENTPD2*
				Differentially expressed genes (n=16)		
Gain (n=5)		Previously identified genes		>2.5 fold increase		*P*<0.0001
12p13.31	·		*NTF3, VWF*		*C3AR1, ACRBP*	
16q12.2	·		·		*CHD9*	
22q11.23	·		*VPREB3, **GSTT1***		·	
3q29	·		*ATP13A3, MUC4*		***PAK2**, LRRC15, MUC4, KIAA0226, ATP13A3, CPN2, **ACAP2***	
8p11.22	·		·		·	

Bold font indicates cancer-related genes according to the Ingenuity knowledge database.

**Figure 5:**
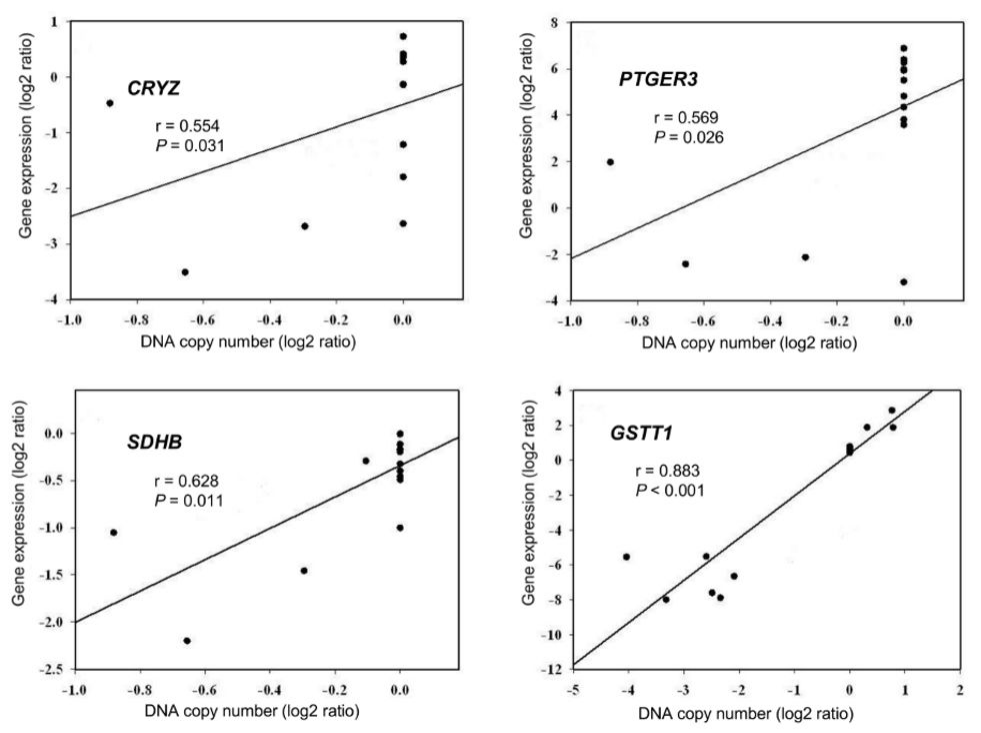
Correlation between DNA copy number and gene expression for CRYZ, PTGER3, SDHB, and GSTT1.

To narrow down the list of candidate genes with expression levels associated with recurrent genetic aberrations, we combined matched CN and expression data in 15 of the 32 cases. After filtering by fold-change (>2.5) and selecting for *P* value (<0.0001), the number of genes that showed altered expression between wild-type/PDGFRA-mutant and KIT-mutant GISTs was reduced to 53 (Table 2). These genes showed either gain and overexpression or loss and underexpression in wild-type/PDGFRA D842V GISTs. Of the genes listed in Tables 1 and 2, eight genes (ACAP2, GSTT1, NRP1, PAK2, SET, SSTR3, TARDBP, and VCAM1) are cancer-related based on the Ingenuity knowledge database (http://www.ingenuity.com), but have not previously been shown to be associated with GISTs. Among them, a significant positive correlation between CNAs and expression was observed for CRYZ (1p31.1), PTGER3 (1p31.2), SDHB (1p36.1-p35), and GSTT1 (22q11.23) (Spearman’s rho > 0.55, *P*<0.05) (Figure 5).

### Increased copy number and mRNA expression of GSTT1 as determined by qRT-PCR

**Figure 6:**
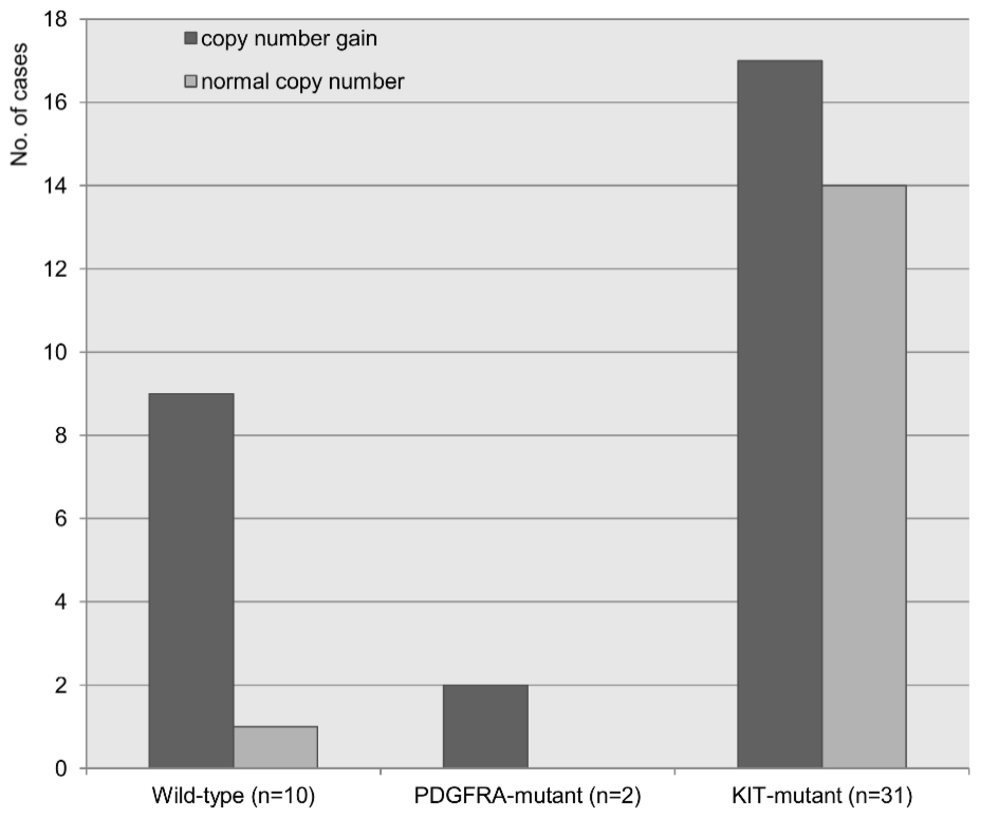
GSTT1 copy number gain detected by quantitative real-time PCR in a validation cohort (n=43).

Our aCGH and expression data showed clear evidence that tumors with higher expression of GSTT1 had CN gains in the chromosomal region corresponding to GSTT1. In addition to the 32 GISTs in this cohort, 11 additional malignant gastric GISTs (six wild-type, one PDGFRA D842V mutant, and four KIT mutants) were tested for CNAs and mRNA expression of the GSTT1 gene by quantitative real-time PCR (qRT-PCR). Gain of GSTT1 CN was demonstrated in 11 of 12 wild-type/PDGFRA D842V GISTs and was significantly more common in these GISTs than in KIT-mutant GISTs (91.7% *vs.* 54.8%; *P*=0.033) (Figure 6). The tumors that showed an increase in GSTT1 CN also expressed higher levels of GSTT1 mRNA than those GISTs without an increase in GSTT1 CN, as determined by qRT-PCR (Spearman’s rho=0.705, *P*<0.001).

To validate the results derived from the gastric GISTs, 11 patients with metastatic small intestinal GISTs (six with KIT exon 11 deletion mutations, one with a KIT exon 13 missense mutation, one with a KIT exon 17 missense mutation, two with KIT exon 9 duplication mutations, and one wild-type GIST) with known clinical outcomes after treatment with imatinib were investigated further. Gain of GSTT1 CN and increased mRNA expression of this gene were observed in all four cases with primary resistance to imatinib despite the presence of KIT exon 11 deletion mutations.

### Loss of heterozygosity in 1p in GISTs with copy number losses at 1p36.33-p11.2

**Figure 7:**
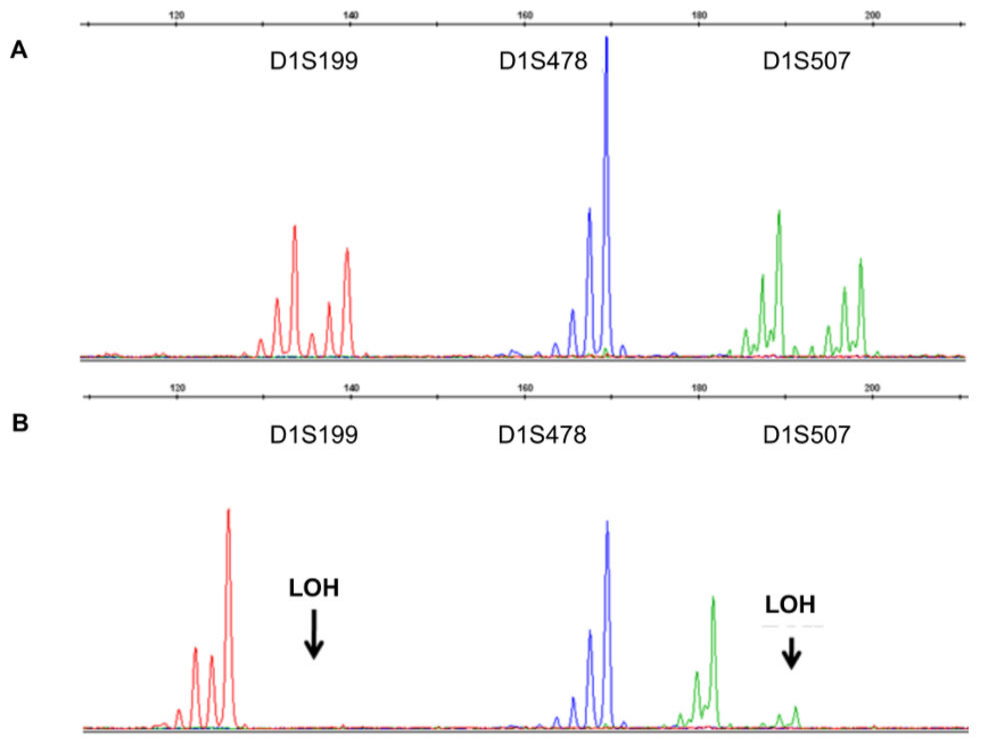
Representative photographs of loss of heterozygosity found on 1p (A, normal; B, tumor).

As LOH has been suggested to be the cause of large deletions of the SDHB gene [19], seven tumors (five with KIT/PDGFRA mutations and two wild-type GISTs) with CN losses at 1p36.33-p11.2 were tested for LOH (Table 3). All tested tumors showed LOH in more than one tested marker (Figure 7).

**Table 3:** Microsatellite analysis in 7 gastric gastrointestinal stromal tumors with copy number loss on 1p36.33-p11.2.

				Microsatellite markers			
Case No.	Gender/Age (yr)	Risk of progression	Detected mutation	D1S199	D1S478	D1S507	Recurrence or metastasis
9	F/52	low	wild-type	■	–	□	no
11	M/66	low	wild-type	■	–	■	no
21	F/47	moderate	KIT exon 11 duplication	■	–	□	no
24	M/72	moderate	PDGFRA exon 18 missense	■	■	▣	no
28	M/63	high	KIT exon 11 deletion	■	–	■	yes
29	M/53	high	KIT exon 11 duplication	■	–	■	no
31	F/63	high	KIT exon 11 insertion	■	–	■	no

■ loss of heterozygosity; □ both alleles retained; ▣ microsatellite instability; - noninformative

## Discussion

Although sequential accumulation of other genetic events besides KIT/PDGFRA mutations is involved in the development and progression of GISTs, the biologic significance and clinical implications of these other genetic events have not been thoroughly investigated [20,21]. Moreover, resistance to imatinib develops over time during treatment, and new therapeutic approaches are needed [1]. To identify additional drivers or modifiers of GIST biology that can be targeted, we integrated array-based analysis of DNA CN and gene expression results from tumors resistant to imatinib, i.e. wild-type and PDGFRA D842V GISTs (summarized in Table S3). This approach enabled us to identify candidate genes underlying resistance to imatinib therapy. All 28 mutant GISTs and 4 wild-type GISTs exhibited CNAs in multiple foci. Two previous studies using SNP arrays showed that wild-type GISTs show few or no CNAs, demonstrating minimal cytogenetic progression [20,22]. However, another study with oligonucleotide microarrays reported deletions in chromosomes 1, 14 and/or 22 in four wild-type GISTs [13]. These discrepancies might be due to technical differences in the studies, and previous CGH studies used formalin-fixed and paraffin-embedded tumor tissues, which may decrease the likelihood of detecting minor alterations [22].

Defects in the SDH complex of respiratory chain complex II have been identified in wild-type GISTs lacking germline mutations in any of the SDH subunit genes [23]. Recently, in addition to germline mutations of SDHB, SDHC, and SDHD genes, loss of function mutations of SDHA have been reported and tumors in affected patients showed either loss or somatic mutation of the remaining wild-type allele [24]. In our study, the expression of all SDH subunits was significantly lower in wild-type/PDGFRA D842V-mutant GISTs than KIT-mutant GISTs. Loss of SDH complex activity in GISTs can result in cytoplasmic accumulation of SDH and lead to increased levels of hypoxia-inducible factor 1α (HIF1α), which activates the transcription of vascular endothelial growth factor (VEGF) and insulin-like growth factor 2 (IGF2) [1]. Previously, high expressions of potential druggable targets, such as VEGF, MCSF and BCL2, were reported in wild-type GISTs [25]. In addition, overexpression of IGF1R has been demonstrated in wild-type GISTs [26,27]. Here, we confirmed that VEGF and IGF1R expression levels were higher in wild-type/PDGFRA D842V-mutant GISTs than KIT-mutant GISTs, further supporting upregulation of the IGF and HIF1α pathways in these tumors. Thus, in patients with wild-type or imatinib-resistant GISTs, attempts to target IGF1R and VEGFR2 would seem to be reasonable options.

Despite the initial efficacy of imatinib in GIST patients, many acquire resistance to this drug, frequently due to secondary mutations in KIT. In addition, the increased expression of ZNF subfamilies has been proposed as an additional mechanism underlying resistance to imatinib in chronic myeloid leukemia (CML) and GIST patients [12,28]. ZNF genes, located within the 19p12-13.1 locus, were expressed at higher levels in our wild-type/PDGFRA-mutant GISTs independent of CNAs. Increased mRNA expression of these genes has been reported in pretreatment biopsy samples from GISTs unresponsive to short-term imatinib treatment [12]. siRNA targeted knockdown of a subset of ZNFs could enhance the sensitivity of GIST cells to imatinib, suggesting these genes are not only predictive of imatinib response, but also have functional relevance to drug activity [12]. A recent study by the same group also demonstrated that knockdown of ZNFs led to downregulation of TGFb3, periostin, and NEDD9 [29]. Other mechanisms of imatinib-resistance in GISTs include pharmacokinetic variability linked to individual metabolic traits and alterations in transporter enzymes [30]. Glutathione S-transferases (GSTs) are a family of enzymes that catalyze the conjugation of glutathione with charged compounds; they function in protecting cells from environmental and oxidative stress [31,32]. Chemotherapeutic-resistant cancer cell lines overexpress GST isozymes, leading to accelerated detoxification of drug substances and drug resistance to compounds targeting the MAPK signaling pathway [32,33]. The mechanisms responsible for GST overexpression include transcriptional activation, stabilization of mRNA and protein, and gene amplification [33]. Recently, GSTT1 CN gain was reported to be associated with a poor response to imatinib dose escalation in patients with CML [34]. In a proteomic study of GISTs, overexpression of a GST isozyme was also observed in wild-type GISTs [35]. For the first time, we identified CN gains at 22q11.23 in GIST samples, and the expression array confirmed overexpression of GSTT1. These observations were validated in clinical samples by qRT-PCR: CN gain of GSTT1 was detected in 90% of wild-type and 100% of PDGFRA D842V GISTs, and all cases with GSTT1 CN gain showed disease progression during imatinib therapy. Moreover, in an independent validation cohort consisting of 11 malignant small intestinal GISTs, all four GISTs with GSTT1 CN gain and increased GSTT1 mRNA expression did not respond to imatinib despite having imatinib-sensitive KIT exon 11 deletion mutations. Our findings strongly indicate that CN gain of GSTT1 may affect the response to imatinib in GISTs, irrespective of mutation status and tumor location, which is a new molecular mechanism of primary resistance and disease persistence during tyrosine kinase inhibitor therapy.

In conclusion, GISTs with CN losses on 1p36.33-p11.2 showed LOH in the SDHB gene. In addition to upregulation of IGF1R and VEGF, frequent CN gain and increased mRNA expression of GSTT1 as well as significant overexpression of ZNF subfamily members were observed in wild-type/PDGFRA D842V GISTs compared to KIT-mutant GISTs. CN gain of GSTT1 was closely associated with imatinib resistance. Based on these findings, analyses of GSTT1 CN and ZNF expression may predict clinical responses to imatinib in GIST patients. Further large-scale and well-designed clinical studies are warranted.

## Supporting Information

Table S1 **Clinicopathologic features and array comparative genomic hybridization results of 32 gastric gastrointestinal stromal tumors.**
(PDF)

Table S2 **Altered gene expressions in wild-type/PDGFRA-mutant gastrointestinal stromal tumors compared to KIT-mutant tumors.**
(PDF)

Table S3 **Summary of molecular analyses for 32 gastric gastrointestinal stromal tumors.**
(PDF)
