# Supplementary material for: GSTT1 Copy Number Gain and ZNF Overexpression Are Predictors of Poor Response to Imatinib in Gastrointestinal Stromal Tumors
Source: PLoS One. 2013 Oct 4;8(10):e77219. doi: 10.1371/journal.pone.0077219 (PMC3790698; doi:10.1371/journal.pone.0077219)
Supplement: Table S1 — Clinicopathologic features and array comparative genomic hybridization results of 32 gastric gastrointestinal stromal tumors. (PDF) [file pone.0077219.s001.pdf]

Table S1. Clinicopathologic features and array comparative genomic hybridization results of 32 gastric gastrointestinal stromal tumors.

Case No.	Gender /Age (yr)	Risk of progression	KIT and PDGFRA genotype	DNA copy number changes	Recurrence or metastasis
1	F / 67	very low	KIT exon 11 missense	-1q21.3, -1q42.13, -2p15, -2p11.2-p11.1, +3p26.3, -3q26.1, +5p15.33, +5p15.33-p11, +5q11.1-q35.3, -5q31.3, -5q35.2, -6p21.33, -6p21.32, -11p15.4, -11q11, -11q23.1, -14q11.2, +14q11.2, -14q24.3, +14q32.33, +15q11.1-q11.2, -16p12.2, -16q22.1, -16q23.1, -17q12, -17q25.3, -22q11.21, -22q11.23, -22q13.2, -Yp11.31-p11.2, -Yp11.2, +Yp11.2, -Yq11.21-q11.23	no
2	M / 68	very low	KIT exon 11 missense	+1p36.33, -1p36.33, -1q21.2, -1q42.13, -2p15, -2q37.3, -3q26.1, -4q13.2, -6p25.3, -6p22.1, -6p21.32, +7p22.3, +7q36.1, +7q36.2, +8p11.22, -8q24.3, -9q34.3, +10q26.3, +11q11, +12p13.2, -13q21.1, -13q21.31, -14q11.2-q32.33, +15q11.1-q11.2, -16p13.11, -16p11.2, -16q12.2, +16q12.2, -16q22.1, -16q23.1, -16q24.2, -17q25.3, +18q22.3-q23, -19p13.3, -20p13, -22q11.21, -22q11.23, -Xp22.33-p11.1, -Xq11.1-q27.1, -Xq21.31-q21.32, -Xq27.1-q28, +Yp11.31, +Yp11.2, +Yq11.21-q11.223	no
3	F / 78	very low	KIT exon 11 missense	+1p21.1, +1q21.3, +1q44, -2q23.3, -3q26.1, +4p11, -4q13.2, -4q31.21, -5q31.3, -6p22.1, +7p22.3, -7q35, +8p11.22, -11p15.5-p11.12, -11q11, -12p12.3, -14q24.3, -14q32.33, +15q11.1-q11.2, -15q13.1-q13.3, -15q14-q26.3, -16p13.11, -16q12.2, +16q12.2, -16q22.1, -16q23.1	no
4	F / 61	very low	KIT exon 11 deletion	-1p36.13, -1p12, -1q21.3, -3q26.1, -4q13.2, -5q13.2, -5q31.3, -6p22.1, -6p21.32, +8p23.2, +8p11.22, -8q12.3, -9p24.3-p11.2, -9q13-q34.3, +11q11, -13q21.31, -14q11.1-q32.33, +15q11.1-q11.2, -15q14, -16p13.11, +16p11.2, -16p11.2, -17q21.32, -17q25.3, +20p13, -22q11.1-q13.33	no
5	M / 70	very low	KIT exon 11 deletion	+1q21.2, +2p13.1, -3q26.1, -4q13.2, -5q31.3, -6p21.32, +8p11.22, +11p15.4, +11q11, +11q25, -13q14.12, +14q11.2, -14q32.33, -15q11.2, -15q15.3, -16p13.11, -16p12.2, -16q12.2, +16q12.2, -16q24.2, -17q21.31, -17q25.3, +19q13.32, -20p13, -Xp11.23, +Xq28	no

6	M / 52	very low	KIT exon 11 deletion	-1p35.1, -1q21.3, -2p11.2, -2q33.1, -3p21.2, -4q28.2, -5q13.1-q13.2, -5q23.2, -5q31.1, +6p21.32, -6q13, -6q21, -6q25.1, -7p11.2, -7q22.3, -7q36.1, +8p23.3-p23.2, +8p23.1, +8p11.22, -9p24.1, -9p13.3, -11p15.4, -11p15.1, -12p13.33, -12p13.31, +12p13.31, -12p12.3, -14q11.1-q32.33, +15q11.1-q11.2, -15q22.31, -16p12.2, +16q12.2, -16q21, -17q12, -17q21.31, -20p13, -21q22.12-q22.13, -22q11.1-q13.33	no
7	M / 35	very low	KIT exon 11 deletion	-1p36.33, +1p36.13, +1q21.3, -1q24.2-q31.1, -1q32.3-q44, -3q21.3, -3q26.1, -3q29, +5p15.33, +5q11.2-q35.3, -6p21.32, +8p11.22, -9q34.3, -10q24.1, -11q11, -12q24.33, -13q34, -14q11.2-q32.33, -16p13.11, -16q22.1, -16q22.2, -16q24.3, -17q21.31, -19q13.42, +20p13, -21q22.3, -22q13.1, -22q13.2	no
8	F / 68	very low	KIT exon 17 missense	-1p36.33, -1p36.21, -1q21.3, -1q32.1, -1q42.13, -1q44, -2q21.1, -2q21.2, -2q37.3, -3p21.31, -3q26.1, -4q13.2, +4q32.2, -5q35.2, -6p24.3, -6p21.33, -6p21.32, +6q27, -7p11.2, -7q11.23, -7q22.1, +8p11.22, +8p11.21, -9p13.1-p11.2, -9q34.2, -9q34.3, -11p15.5, -11p11.12, -11q11, -12p13.31, -12q13.12, -13q12.11, +14q11.2, -14q24.3, -14q32.33, -15q11.1-q11.2, -15q13.3, -15q24.3, -15q26.1, -16p13.3, +16q12.2, -16q24.2, +17p13.3, -17p13.1, -17q11.2, -17q25.3, -18q21.1, -19p13.3, -19p13.11, -19q13.12, -19q13.31, -19q13.42, -19q13.43, -20p13, -20q11.22, -21q11.2, -21q22.11, -22q11.21, -22q11.23, -22q13.1, -Xp22.31, -Xp11.23, -Xp11.22, -Xq21.31-q21.32, -Xq28, -Yp11.31-p11.2, +Yp11.2, -Yq11.21-q11.23	no
9	F / 52	low	wild-type	-1p36.33-p11.2, +1p36.13, -1q21.2, -1q21.3, -1q25.2, -1q42.13, -2q21.1, +2q21.2, -2q37.3, -3p25.2, -3q26.1, +3q29, -4q13.2, +4q32.2, +5p15.33, +5q35.2, -6p22.1, +6p21.32, -6q21, -7q11.23, -7q35, +8p11.22, -9p13.1-p11.2, -9q21.32, -9q34.3, -10q23.2, -11p15.5, -11q11, -11q22.3, -12p13.33, -12p13.31, +12p13.31, -12q13.12, -14q13.1-q13.2, -14q32.33, +15q11.1-q11.2, -15q14, -16p13.11, +16q12.2, -16q24.2, -17q11.2, +17q12, -17q21.32, -17q25.3, -18q21.1, -19p13.3-p12, -19q11-q13.2, -19q13.31-q13.43, -20p13, -21q22.11, -22q11.1-q13.33, -Xp11.22, -Xq28, -Yp11.31-p11.2, +Yp11.2, -Yq11.21-q11.223, -Yq11.23	no
10	M / 75	low	wild-type	+1p36.13, +1q21.2, -1q42.13, -3q26.1, -3q29, -4q13.2, -6p21.32, -8p23.1, +8p11.22, -8q23.3, -9q34.3, +12p13.31, -14q11.2-q32.33, -16p13.11, -16q22.1, -16q23.1, -17q21.31, -17q25.3, +20p13, +22q11.23	no

11	M / 66	low	wild- type	-1p36.33-p11.2, +1q21.2, -1q21.3, -1q31.3, -1q32.1, -1q42.13, -2p23.3, -2p22.2-p22.1, -2p16.1-p15, -2q37.3, -3q12.3, -3q26.1, +3q29, -4p14, -4q13.2, -4q13.3, -4q32.3, -5q13.1-q13.2, -5q31.1, -5q31.2, -6p21.32, -6p21.31, -6q21, -7q11.23, +8q24.3, -9q21.32, -9q34.3, -10p11.22, -11p15.5, -11p15.1, +11q14.3, -11q23.3, -12p13.31, -12p12.1, +14q11.2, -14q13.1-q13.2, -14q24.3, -14q32.33, -15q11.1, -15q11.2-q26.3, -16p13.3, +16q12.2, -16q23.2, -16q24.2, -17q11.2, +17q21.32, -17q25.3, -18q21.1, -19p13.3-p12, -19q11-q13.43, -20p13, -21q22.11, -22q11.21, -22q11.23, +22q11.23-q12.1, -Xp22.33, -Xq24, -Xq28, -Yp11.2, -Yq11.23	no
12	F / 34	low	wild-type	-1p13.3, -1q21.3, -3q26.1, +3q29, -4q13.2, +5p15.33, -6p21.33, -6p21.32, -7q35, +8p23.1, +8p11.22, -11p15.4, +11q11, +12p13.31, -13q21.1, -13q34, -14q24.3, -14q32.33, +15q11.1-q11.2, -16p13.11, -16q12.2, -16q22.1, -16q23.1, -17q21.31, -17q25.3, -19q13.31, +20p13, +22q11.23	no
13	M / 55	low	KIT exon 11 missense	-1q31.3, +1q44, -1q44, -3p21.2, -3q26.1, -4q13.2, -5q13.1-q13.2, -6p21.32, -6q13, +7q35, -8p22, +12p13.31, -14q11.2-q32.33, +15q11.1-q11.2, -16p13.11, -16p12.2, -17q21.31, -22q13.1, +Yq11.222	no
14	M / 65	low	KIT exon 11 missense	+1q21.3, -1q43, -2q21.1, -2q33.1, -3p25.3, -3q26.1, -3q26.2, -4p14, -4q32.3, +5p15.33, -5q12.1, -5q31.1, +5q35.3, -6p21.32, -6q13, -6q15, -6q21, -7q36.1, -8p12, +8p11.22, -8q13.1, -8q13.3, -9p24.1, -9p12-p11.2, +9q34.2, +11p15.5, -11p15.1, -11q11, -11q22.3, -12p13.33, -12p13.31, -12q23.1, -12q24.33, -13q12.11, -13q34, -14q11.2-q32.33, -15q11.2, -15q14, -15q22.31, -16p13.11, +16p11.2, -16q23.1, -17p11.2, -17q21.31, +17q21.31, +17q21.32, -18p11.32, -19p13.3, +20p13, -21q22.12-q22.13, -21q22.2, -22q11.23, -Xp11.23	no
15	M / 45	low	KIT exon 11 missense	+1p36.33-p11.2, +1q21.1-q44, -1q21.3, -1q44, +2p25.3-p11.1, +2q11.1-q37.3, -3q21.3, -3q26.1, +3q29, +4p16.3-p11, +4q11-q13.2, -4q13.2, +4q13.2-q35.2, +5p15.33-p11, +5p13.2, +5q11.1-q35.3, +6p21.32, +6p12.1, -6q13, +7p22.3-p11.2, +7q11.21-q36.3, +8p23.3-p11.1, +8q11.1-q24.3, +9p24.3-p11.2, +9q13-q34.3, +11p15.5-p11.12, +11q11-q25, +12p13.33-p11.1, +12q11-q24.33, +12q24.23-q24.31, +13q11-q34, -14q11.2, +14q11.2, -14q24.3, +14q32.33, +15q11.1-q11.2, +16p13.3-p11.1, -16p13.11, +16q11.2-q24.3, -16q22.1, -16q23.1, +17p13.3-p11.2,	no

				+17q11.1-q25.3, +17q25.1, +17q25.3, +18p11.32-p11.21, +18q11.1-q23, +19p13.3-p12, +19q11-q13.43, -19q13.2, +20p13-p11.1, +20q11.21-q13.33, +21p11.2-p11.1, +21q11.2-q22.12, -21q21.3, +21q22.13-q22.3, -Xp11.4, -Xp11.23, -Xq24	
16	M / 56	low	KIT exon 11 duplication	+1p34.2, -1q21.3, -2q33.3, -3q26.1, -4q13.2, +5p15.33-p11, +5q11.1-q35.3, +6p21.33, -6p21.32, +6p21.32, -6q13, -6q21, -7p11.2, -7q32.2, -7q36.1, +8p23.3-p11.1, +8q11.1-q24.3, -11p15.1, -12p13.31, -12q13.3, -14q11.2, +14q32.33, -14q32.33, +15q11.1-q11.2, -15q15.1, +16p11.2, +17p13.3-p11.1, +17q11.1-q25.3, -17q21.31, +17q21.32, +18q11.1-q23, +20p13, -21q22.12-q22.13, -22q11.1-q13.33, +Xq13.2	no
17	M / 73	moderate	KIT exon 11 missense	-1p13.3, -1q21.2, -1q21.3, -2p11.2, -4q13.2, -4q28.1-q28.2, -4q32.3, -5q13.1-q13.2, -5q23.2, -5q32, -6p21.32, -6q13, +7p22.3, -7q36.1, +8p23.1, -8q24.21, -9q33.3, -11p15.1, +11q11, -12p13.33, -13q31.3, -14q11.2-q32.33, -15q11.1-q11.2, +16p11.2, -16q12.2, +16q12.2, -16q24.2, -17q21.31, +20p13, -22q11.1-q13.33, -Xp22.11	no
18	M / 77	moderate	KIT exon 11 missense	-1p36.21, -1q21.2, +1q21.3, -1q44, -3p14.1-p13, -3q26.1, -3q29, -4p14, +6p21.32, -7p21.2, +8p11.22, -10p15.1, -14q11.2-q32.33, -15q11.1-q11.2, -15q15.3, -16p13.11, -16p11.2, -17q21.31, +20p13, -22q11.23, -22q13.1	no
19	F / 76	moderate	KIT exon 11 missense	+1p21.1, -2q37.3, -3p26.3-p12.2, -3q11.1-q22.1, -3q26.1, -4q13.2, -6p22.1, +7q34, -9p12-p11.2, +10p11.23, +11q11, -13q21.1, -13q22.3-q34, -14q11.2-q31.2, -14q32.33, -16p13.11, +20p13, -22q13.1, +Yp11.2	no
20	F / 81	moderate	KIT exon 11 deletion	-1q21.3, -3q26.1, -3q29, -4q13.2, +5p15.33, -6p22.1, +6p12.1, +7q34, +8p11.22, -14q11.2-q32.33, +15q11.1-q11.2, +16p11.2, +17q21.31, -22q11.1-q13.33	no
21	F / 47	moderate	KIT exon 11 duplication	-1p36.33-p11.2, +1p36.21, -1q21.1-q44, -2q13, -3q26.1, -4p16.3-p11, -4q12-q35.2, -6p25.3-p11.2, -6q11.1-q27, -7p22.3-p11.2, -7q11.21-q36.3, +7q34, -8p23.1, +8p11.22, -9p12-p11.2, -10p15.3-p11.1, -10q11.21-q26.3, +11q11, -14q11.2-q32.33, -14q24.3, -14q32.33, +15q11.1-q11.2, -15q11.2-q26.3, -16p13.11, -16q22.1, -17q12, -17q25.3, -19q13.2-q13.31, -19q13.43, -21p11.2-p11.1, -21q11.2-q22.3, -22q11.1-q13.33, -Xq13.3-q21.1, -Yp11.2	no

22	F / 52	moderate	KIT exon 11 deletion	+1q21.3, -1q24.2, -4q13.2, -4q35.2, +5p15.33, -6p25.3, -6p21.32, -6q13, -9p12-p11.2, -9q21.11, +11q11, -14q11.2-q32.33, -16p13.11, -16q23.1, +Xq21.32	no
23	F / 69	moderate	KIT exon 11 deletion	+1q21.3, +1q32.2, -1q44, -3q26.1, +5p15.33-p12, +5q11.1-q35.3, -6p22.1, -6p21.32, +7q34, +8p23.3-p11.1, +8q11.1-q24.3, +9p24.3-p11.2, +9q13-q34.3, -9q34.3, +11q11, -13q21.1, +14q11.2, -14q24.3, -14q32.33, +15q11.1-q11.2, -16p13.3, -16q12.2, +16q12.2, -16q24.1, +17p13.3-p11.2, +17q11.1-q25.3, -20p13, -22q11.23-q12.1, -22q13.1, +Yp11.2, -Yq11.21-q11.223	no
24	M / 72	moderate	PDGFRA exon 18 missense	-1p36.33-p11.2, -1q21.1, +1q21.3, +2q37.1, +3p26.3, -4p16.3-p11, +4q32.2, -6p21.32, +7q35, -11p15.4, -11q11, +12p13.31, -14q11.2-q32.33, +16p13.3, -16q12.2, +16q12.2, -16q22.1, -17q21.31, -20p13, +20q13.13	no
25	F / 53	high	KIT exon 11 missense	-1p36.33, -1p36.32-p36.31, -1p36.22, +1p36.21, -1p36.21, -1q42.13, -2p25.3, -2p15, +2q21.2, -2q37.3, -3q12.3, -3q26.1, -4q13.2, -4q23, -5q13.1-q13.2, -5q35.3, -6p22.1, -6p21.32, -6q21, -7p22.1, +7p21.3, -7p11.2, -7q11.23, -7q36.1-q36.2, -8p23.3-p11.1, +8p11.22, +8q11.1-q24.3, -8q24.3, -9p21.3, -9p13.1-p11.2, -9q13-q31.2, -9q34.3, -10p15.3-p11.1, -10q24.32, -10q25.1-q26.3, -11p15.5-p13, -11p12-p11.12, -12p13.33, -12p13.31, -12q24.31, -14q11.2-q32.33, -15q11.1, -15q24.3, -16p13.11, -16p12.2, +16p11.2, -16q12.2, +16q12.2, -16q22.1, -16q24.2, -17q11.2, -17q12, -17q25.3, -18q21.1, -19p13.3, -20p13, -21q11.2, -21q22.11, -21q22.2, -22q11.23, -Xp22.33-p11.1, -Xq11.1-q28, -Yp11.31-p11.2, +Yp11.2, -Yq11.21-q11.23	no
26	M / 66	high	KIT exon 11 missense	-1p36.33, +1p36.13, -1q42.13, +1q44, -1q44, -2q12.2, +2q37.1, -2q37.3, -3p25.2, -3p21.31, -3q26.1, -4q13.2, -6p21.33, -6p21.32, +8p23.1, +8p11.22, -8q13.1, -9q34.3, -11p15.4, +11q11, -12p13.33, -12p13.31, -14q11.2-q32.33, +15q11.1-q11.2, +15q15.3, -16p13.11, +16q12.2, -16q22.1, -16q23.1, -16q24.2, -17q21.31, -17q25.3, -19p13.3, -21q22.11, -21q22.12-q22.13, -22q11.23, -Xq28, -Yp11.2	loss of follow-up
27	M / 60	high	KIT exon 11 deletion	+1p36.33-p11.2, -1p36.21, -1p13.3, +1q21.1-q44, -1q21.3, +2p25.3-p11.1, +2q11.1-q37.3, -3q26.1, -4q13.2, -4q32.3, -4q35.1-q35.2, +5p15.33-p11, +5q11.1-q35.3, -5q13.2, +6p25.3-p11.2, -6p21.32, +6q11.1-q27, +7p22.3-p11.2, +7q11.21-q32.1, -7q34,	yes

				+7q36.2-q36.3, +8p23.3-p11.1, +8q11.1-q24.3, +11p15.5-p11.12, +11q11-q25, +12p13.33-p11.1, +12q11-q34.33, +13q11-q34, +14q11.2, -14q24.3, +14q32.33, -14q32.33, +15q11.1-q26.3, +16p13.3-p11.1, -16p13.11, +16q11.2-q24.3, -16q24.2, +17p13.3-p11.2, +17q11.1-q25.3, +19p13.3-p12, +19q11-q13.43, +20p13-p11.1, -20p13, +20q11.21-q13.33, +21p11.1, +21q11.2-q22.3, -22q11.23, -Xq22.3, +Xq27.1, +Xq27.3-q28	
28	M / 63	high	KIT exon 11 deletion	+1p36.33-p36.32, -1p36.32-p36.22, -1p36.22-p34.3, +1p34.3-p11.2, +1q21.1-q44, -1q44, -2q37.3, -3p26.2-p26.1, -3p24.3-p24.1, -3p22.2-p21.31, -3p21.2-p14.3, -3q26.1, -4p14-p13, -4q13.2, -5p13.2, -6p25.3, +6p21.32, -7q31.1, +7q35, -8p23.3-p11.1, +8q11.1-q24.3, -9q22.32, -9q33.3-q34.3, +10q11.22, +11p15.5-p11.12, +11q11-q12.3, +11q13.2-q25, +12p13.31, +13q11-q12.11, -13q14.3-q21.31, -13q21.32-q22.3, -13q31.1-q34, -14q11.2-q32.12, -14q32.33, -15q11.2, +16p13.3-p11.1, -16p13.11, +16q11.2-q24.3, -17q21.31, +17q21.31, -22q11.23	yes
29	M / 53	high	KIT exon 11 duplcation	-1p36.33, -1p36.22, -1p36.21-p36.13, -1p36.11-p35.3, -1p35.2-p35.1, -1p32.3, -1p31.1, -1q21.2-q21.3, +1q21.3, -1q25.2, -1q32.1, -1q42.11-q42.12, -1q42.3, -1q44, +1q44, -2p23.1-p22.3, -2p22.2-p22.1, -2p21, -2p16.2, -2p16.1-p15, -2p13.3, -2q14.2-q14.3, -2q33.1, -2q33.3, -2q36.1, -2q36.3, -3p26.3-p11.1, -3q11.1-q29, -3q13.13, -3q21.2, -3q21.3, -3q22.3, -3q23, -3q26.1, -3q26.33-q27.3, -3q29, -4p14, -4q13.2, -4q13.3, -4q21.1, -4q25, -4q28.1-q28.2, -4q31.1, +5p15.33-p12, +5q11.1-q23.3, +5q31.1-q35.2, +5q35.3, -6p25.3-p11.2, -6q11.1-q27, +7p22.2, +7p22.1-p11.2, +7q11.22, +7q11.23-q21.3, +7q22.1-q32.1, +7q32.2-q33, +7q34-q36.3, -8p12, +8p11.22, -8p11.21-p11.1, -8q11.23, -8q12.1, -8q13.1, -8q13.3, -8q21.13, -8q22.1-q22.2, -8q24.12, -9p22.1, -9p13.3, -9q21.32, -9q22.32-q22.33, -9q33.3, -9q34.11, -10p15.3-p11.1, -10q11.21-q26.3, -11p15.4, -11p15.1, -11p11.2, -11q13.4, -11q22.3, -11q23.3, -12p13.33, -12p13.31, -12p12.3, -12q13.11-q13.13, -12q13.2-q13.3, -12q14.2-q14.3, -12q23.1, -12q24.11-q24.13, -12q24.23-q24.31, -12q24.33, -13q14.12-q14.13, -13q21.1, -13q32.1, -13q34, -14q11.2-q32.33, +14q32.33, -15q11.1-q26.3, -16p13.13, -16q22.1, -16q22.2, -16q23.1, -16q23.2, -17p13.3-p13.2, -17q11.2, -17q12, -17q21.2-q21.31, -	no

				17q22-q23.1, -17q24.2, -17q25.1, -18p11.31, -18q11.2, -18q12.1, -18q21.1, -18q21.2, +19p13.2, +19q11-q13.11, -19q13.2, +19q13.32, -19q13.33, -19q13.42, +19q13.43, -20q11.21, -20q13.2, +21q11.2-q22.12, +21q22.13-q22.3, +22q11.22-q11.23, -22q12.1, -22q12.2-q12.3, -22q13.1-q13.2, -Xp22.33-p11.1, -Xq11.1-q28, +Yp11.31-p11.2, +Yq11.21-q11.23	
30	F / 58	high	KIT exon 11 insertion	-3q26.1, -4q13.2, +7q34, -14q11.2-q32.33, -16q12.2, +20p13, +22q11.23, -Xp22.33-p11.1, -Xq11.1-q28	no
31	F / 63	high	KIT exon 11 insertion	-1p36.33-p11.2, +1q21.3, -3q26.1, +5p15.33, -6p21.32, -6q12-q27, +7q34, +11q11, -12p12.3, -12q24.22-q24.33, -13q12.11, -14q11.2-q32.33, -15q22.31, -16q23.1, -17q21.31, -18p11.32-p11.21, -19p13.11-p12, -19q11-q13.43, +20p13, -22q11.22-q12.2, -22q11.23, -22q12.2-q13.33	no
32	M / 76	high	KIT exon 11 deletion	+1q21.3, -1q31.1, -2q37.1-q37.2, -2q37.3, -3p26.3-p24.3, -3q26.1, +3q29, -4q13.2, -6p21.32, +7p22.3-p11.2, +7q21.3-q22.3, +7q31.1-q36.3, +8p23.3-p11.1, +8q11.1-q21.2, -8q21.2, -8q21.3-q24.12, -9p21.3-p11.2, -9q13-q33.1, -9q33.3, -11p15.4, -11q11, -12q24.33, -13q21.1, -13q34, -14q11.2-q32.33, +15q11.1-q11.2, -16p13.11, -16q12.2, +16q12.2, -17q21.31, -19q13.31, -20p13, -21q11.2-q22.3, -22q11.23, +22q11.23-q12.1, -22q13.1, -Xp21.1, +Yp11.2	yes
