# Supplementary material for: GSTT1 Copy Number Gain and ZNF Overexpression Are Predictors of Poor Response to Imatinib in Gastrointestinal Stromal Tumors
Source: PLoS One. 2013 Oct 4;8(10):e77219. doi: 10.1371/journal.pone.0077219 (PMC3790698; doi:10.1371/journal.pone.0077219)
Supplement: Table S2 — Altered gene expressions in wild-type/PDGFRA-mutant gastrointestinal stromal tumors compared to KIT-mutant tumors. (PDF) [file pone.0077219.s002.pdf]

Table S2. Altered gene expressions in wild-type/PDGFRA-mutant gastrointestinal stromal tumors compared to KIT-mutant tumors.

**Genes**	**Location**	**Fold change**	***P* value**
BRAF	7q34	0.50	0.001
NF1	17q11.2	1.96	<0.001
SDHA	5p15	-1.18	0.005
SDHB	1p36.1-p35	-0.96	0.041
SDHC	1q23.3	-3.23	<0.001
SDHD	11q23	-0.85	0.025
VEGF	6p12	2.31	0.025
IGF1R	15q26.3	2.76	0.062
MYC	8q24.21	2.21	0.017
MDM2	12q14.3-q15	2.54	<0.001
CCND1	11q13	2.67	0.002
ETV1	7p21.3	-2.18	0.006
mTOR	1p36.2	-0.48	0.035
STAT3	17q21.31	-0.64	0.004
