# Supplementary material for: GSTT1 Copy Number Gain and ZNF Overexpression Are Predictors of Poor Response to Imatinib in Gastrointestinal Stromal Tumors
Source: PLoS One. 2013 Oct 4;8(10):e77219. doi: 10.1371/journal.pone.0077219 (PMC3790698; doi:10.1371/journal.pone.0077219)
Supplement: Table S3 — Summary of molecular analyses for 32 gastric gastrointestinal stromal tumors. (PDF) [file pone.0077219.s003.pdf]

Table S3. Summary of molecular analyses for 32 gastric gastrointestinal stromal tumors.

Case No.	Array CGH	Gene expression array	qRT-PCR (GSTT1)	LOH analysis (SDHB)	Gender	Age (yr)	Risk of progression	KIT/PDGFRA genetype
1	○		○		F	67	very low	KIT exon 11 missense
2	○	○	○		M	68	very low	KIT exon 11 missense
3	○	○	○		F	78	very low	KIT exon 11 missense
4	○		○		F	61	very low	KIT exon 11 deletion
5	○	○	○		M	70	very low	KIT exon 11 deletion
6	○		○		M	52	very low	KIT exon 11 deletion
7	○	○	○		M	35	very low	KIT exon 11 deletion
8	○	○	○		F	68	very low	KIT exon 17 missense
9	○	○	○	○	F	52	low	wild-type
10	○	○	○		M	75	low	wild-type
11	○		○	○	M	66	low	wild- type
12	○	○	○		F	34	low	wild-type
13	○		○		M	55	low	KIT exon 11 missense
14	○		○		M	65	low	KIT exon 11 missense
15	○		○		M	45	low	KIT exon 11 missense
16	○		○		M	56	low	KIT exon 11 duplication
17	○		○		M	73	moderate	KIT exon 11 missense
18	○		○		M	77	moderate	KIT exon 11 missense
19	○		○		F	76	moderate	KIT exon 11 missense
20	○		○		F	81	moderate	KIT exon 11 deletion
21	○		○	○	F	47	moderate	KIT exon 11 duplication
22	○		○		F	52	moderate	KIT exon 11 deletion
23	○		○		F	69	moderate	KIT exon 11 deletion

24	○	○	○	○	M	72	moderate	PDGFRA exon 18 missense
25	○	○	○		F	53	high	KIT exon 11 missense
26	○	○	○		M	66	high	KIT exon 11 missense
27	○	○	○		M	60	high	KIT exon 11 deletion
28	○		○	○	M	63	high	KIT exon 11 deletion
29	○		○	○	M	53	high	KIT exon 11 duplcation
30	○	○	○		F	58	high	KIT exon 11 insertion
31	○	○	○	○	F	63	high	KIT exon 11 insertion
32	○	○	○		M	76	high	KIT exon 11 deletion
